# Circadian Control of the Daily Plasma Glucose Rhythm: An Interplay of GABA and Glutamate

**DOI:** 10.1371/journal.pone.0003194

**Published:** 2008-09-15

**Authors:** Andries Kalsbeek, Ewout Foppen, Ingrid Schalij, Caroline Van Heijningen, Jan van der Vliet, Eric Fliers, Ruud M. Buijs

**Affiliations:** 1 Netherlands Institute for Neuroscience, Amsterdam, The Netherlands; 2 Department of Endocrinology and Metabolism, Academic Medical Center (AMC), University of Amsterdam, Amsterdam, The Netherlands; 3 Instituto de Investigaciones Biomedicas UNAM, Ciudad Universitaria, Mexico City, Mexico; University of Las Palmas de Gran Canaria, Spain

## Abstract

The mammalian biological clock, located in the hypothalamic suprachiasmatic nuclei (SCN), imposes its temporal structure on the organism via neural and endocrine outputs. To further investigate SCN control of the autonomic nervous system we focused in the present study on the daily rhythm in plasma glucose concentrations. The hypothalamic paraventricular nucleus (PVN) is an important target area of biological clock output and harbors the pre-autonomic neurons that control peripheral sympathetic and parasympathetic activity. Using local administration of GABA and glutamate receptor (ant)agonists in the PVN at different times of the light/dark-cycle we investigated whether daily changes in the activity of autonomic nervous system contribute to the control of plasma glucose and plasma insulin concentrations. Activation of neuronal activity in the PVN of non-feeding animals, either by administering a glutamatergic agonist or a GABAergic antagonist, induced hyperglycemia. The effect of the GABA-antagonist was time dependent, causing increased plasma glucose concentrations only when administered during the light period. The absence of a hyperglycemic effect of the GABA-antagonist in SCN-ablated animals provided further evidence for a daily change in GABAergic input from the SCN to the PVN. On the other hand, feeding-induced plasma glucose and insulin responses were suppressed by inhibition of PVN neuronal activity only during the dark period. These results indicate that the pre-autonomic neurons in the PVN are controlled by an interplay of inhibitory and excitatory inputs. Liver-dedicated *sympathetic* pre-autonomic neurons (responsible for hepatic glucose production) and pancreas-dedicated pre-autonomic *parasympathetic* neurons (responsible for insulin release) are controlled by inhibitory GABAergic contacts that are mainly active during the light period. Both sympathetic and parasympathetic pre-autonomic PVN neurons also receive excitatory inputs, either from the biological clock (sympathetic pre-autonomic neurons) or from non-clock areas (para-sympathetic pre-autonomic neurons), but the timing information is mainly provided by the GABAergic outputs of the biological clock.

## Introduction

The role of the CNS in glucoregulation has been recognized since the classical experiments of Bernard in 1849 [1]. The hypothalamus is considered the crucial CNS region involved in glucose metabolism, using a rich innervation of the liver by sympathetic and parasympathetic nerves to regulate glucose metabolism in a reciprocal manner [2]–[4]. The hypothalamus also harbors the endogenous or biological clock in the suprachiasmatic nuclei (SCN) [5]–[7]. The biological clock imposes a temporal structure on the brain and peripheral organs via both neural and endocrine outputs [8], [9]. Previously, we proposed that the biological clock generates the daily rhythm in plasma melatonin concentrations via the combination of a continuous (glutamatergic) stimulation of the pre-autonomic neurons in the paraventricular nucleus of the hypothalamus (PVN) that are at the origin of the sympathetic innervation of the pineal, and a nocturnal withdrawal of the inhibitory (GABA-ergic) SCN inputs to these neurons [10], [11]. To further investigate SCN control of autonomic nervous activity we focused in the present study on the daily rhythm in plasma glucose concentrations, especially in view of the recently demonstrated rhythmic control of glucose metabolism in the liver [12]–[15] and the clear involvement of the sympathetic and parasympathetic input to the liver in glucose metabolism. Indeed a daily rhythm in plasma glucose concentrations, with peak levels attained at the end of the sleep period, has been shown previously [16]. Data obtained from both human and rat studies strongly suggest that the rise in plasma glucose concentrations at the end of the sleep period is endogenous and primarily caused by an increased hepatic glucose production (HGP) controlled by the endogenous clock located in the central nervous system [17]–[20]. Moreover, we recently found that local administration of the GABA-A receptor antagonist bicucilline (BIC) or the glutamate receptor agonist NMDA in the PVN caused pronounced hyperglycemia. In both conditions this hyperglycemic effect of PVN stimulation could be prevented by a selective denervation of the sympathetic autonomic input to the liver [21]. By combining the ICV administration of BIC with a euglycemic clamp Lang et al [22] clearly showed that the BIC induced hyperglycemia is due to an increased HGP.

To investigate possible daily changes in the activation of the pre-autonomic PVN neurons, we first compared the hyperglycemic response evoked by GABAergic and glutamatergic (ant)agonists when administered into the PVN at different moments of the L/D-cycle. Secondly we studied the effect of an SCN-lesion, i.e., removal of the proposed GABAergic projection, on the hyperglycemic effect of BIC applied in the PVN. Thirdly, we inhibited neuronal activity in the PVN during a scheduled meal at different times of the light/dark-cycle in order to investigate whether the parasympathetic input to the pancreas, too, is dependent on (daily changes in) PVN neuronal activity. The results are synthesized into a model explaining how the biological clock is able to somatotopically enforce differentiated autonomic rhythms onto different body compartments.

## Results

A total of 280 Wistar rats were used in this study. Histological analysis of probe placement showed that in the majority of the animals, the tip of the microdialysis probes was consistently positioned within 50–100 µm of the borders of the PVN. Typical examples of probe placements can be found in our previous papers [11], [23]. Approximately 20% of the data had to be discarded because of incorrect probe placements, a blockade of the microdialysis probes, a blockade of the jugular catheter, non-sufficient recovery of the animal from the operation, or an incomplete data set.

### Experiment 1: Time dependent effects of daily changes in GABAergic and glutamatergic PVN inputs on basal plasma glucose and insulin concentrations

In order to investigate possible daily changes in the activity of GABA- and/or glutamate-containing projections to the PVN we used muscimol (MUS) and NMDA to activate and bicucilline (BIC) and MK801 to antagonize GABAergic and glutamatergic receptors, respectively, and circulating plasma glucose and plasma insulin as the main readout. Mean basal plasma glucose and plasma insulin concentrations at the start of the different experiments, as well as a statistical analysis of their response are indicated in Tables S1 and S2.

**Figure 1:**
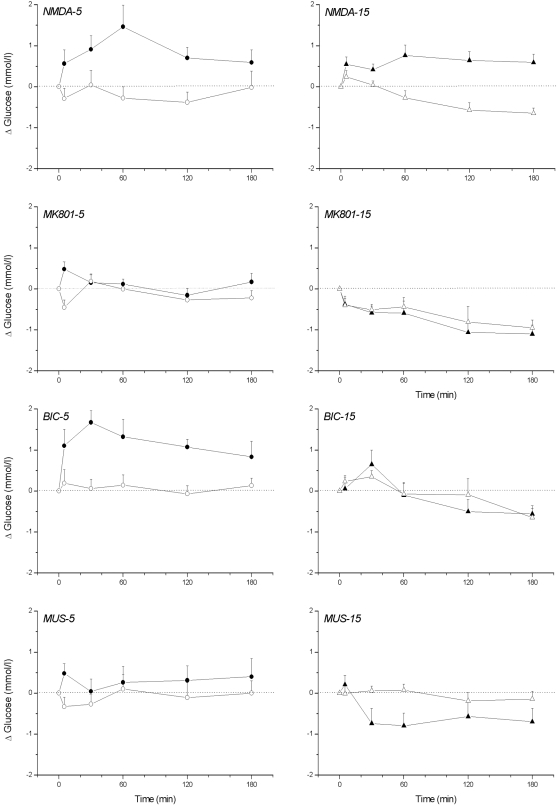
Changes in plasma glucose concentrations during a 2-h administration of NMDA, the NMDA antagonist MK801, Bicuculline, or Muscimol in the PVN at either ZT5 (left column) or ZT15 (right column). Filled symbols indicate the effect of the drug, whereas open symbols show the results of the control experiment in the same animals one week later. Only 3 out of the 8 different treatment protocols showed a significant effect of drug treatment, i.e. NMDA at ZT5 (p = 0.008) and at ZT15 (p = 0.009), and the Bicuculline treatment at ZT5 (p = 0.002).

Only BIC administration showed a strong time-of-day dependency when comparing for all 8 experiments (i.e. BIC, MUS, NMDA and MK801 at either ZT5 or ZT15) the day of drug administration with the control day 1 week later (Fig. 1), i.e., ANOVA showed a significant effect of *Treatment* during the ZT5, but not the ZT15, experiment. For the other drugs either no significant effect of *Treatment* was found, or, as for NMDA, a significant effect of *Treatment* was found at both ZT5 and ZT15.

**Figure 2:**
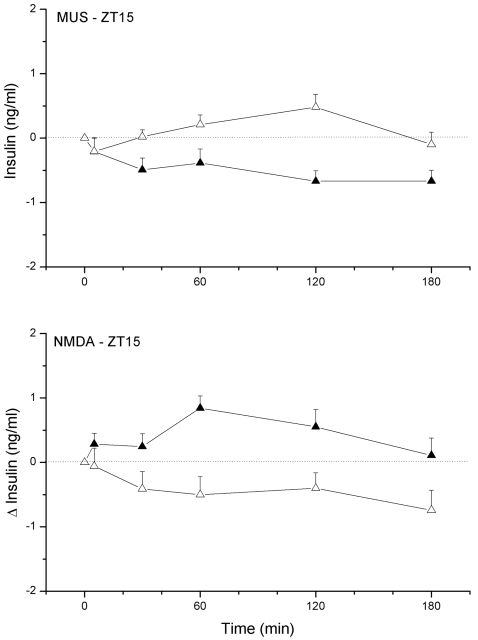
Changes in plasma insulin concentrations during a 2-h administration of NMDA or Muscimol in the PVN at ZT15. Filled symbols indicate the effect of the drug, whereas open symbols show the results of the control experiment in the same animals one week later.

Plasma insulin concentrations were only significantly affected by the ZT15 NMDA and MUS administration (Fig. 2). NMDA administration in the PVN area caused a small increase of plasma insulin concentrations as compared to the control day (p = 0.015 and p = 0.012, effects of *Treatment* and *Interaction*, respectively), whereas administration of MUS caused a significant decrease of plasma insulin concentrations. Basal plasma insulin at ZT15 were significantly lower than those at ZT5, probably due to the 2-h fast at the beginning of the dark period. In conclusion, the time dependency of the BIC-induced hyperglycemia indicates a daily change in GABAergic input to the PVN, with higher levels of GABAergic input during the light period. Especially, since the hyperglycemic effect of NMDA administration in the PVN was present during both the ZT5 and ZT15 experiment it is most probable that the time-dependency of the BIC-induced hyperglycemia is due to a daily rhythm in the activity of the GABAergic inputs to the PVN, instead of daily changes in the pathway downstream from the PVN. Basal plasma insulin levels (i.e. during non-feeding conditions) only respond to changes in PVN neuronal activity during the dark period.

### Experiment 2 : Origin of the daily change in GABAergic input to the PVN

**Figure 3:**
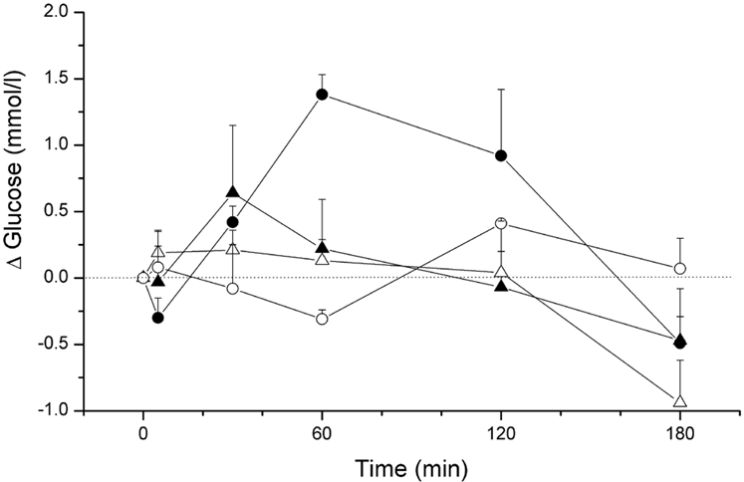
Changes in plasma glucose concentrations during a 2-h administration of Bicuculline in the PVN of SCN-lesioned (closed triangles) and sham-lesioned animals (closed circles). Open symbols show the results of the control experiment in the same animals one week later. Ablation of the SCN caused a profound reduction of the Bicuculline induced hyperglycemia, i.e. there was no significant difference between the Bicuculline treatment and the control day (*Treatment*, p = 0.647; *Sample*×*Treatment*, p = 0.121), contrary to the clear Bicuculline effect in the sham-lesioned animals (*Treatment*, p = 0.013; *Sample*×*Treatment*, p = 0.001). Basal plasma glucose concentrations (at t = 0) did not differ between SHAM-lesioned and SCN-lesioned animals (SCN-Sham: 6.6±0.2 and 6.8±0.3, and SCN-Lesion: 6.7±0.2 and 6.5±0.1 mmol/l for experimental and control days, respectively).

In order to investigate whether the daily change in GABAergic input to the PVN is derived from the biological clock located in the SCN we repeated the PVN administration of BIC in groups of SCN-lesioned and SHAM-lesioned animals. By measuring their diurnal water intake pattern 11 of the 36 animals originally operated upon were identified as having an effective SCN lesion and selected for further experiments. Of the 11 SCN-lesioned and 11 SHAM animals equipped with bilateral PVN probes and a jugular vein catheter, 10 SCN-lesioned animals and 9 SHAM operated animals completed the BIC administration experiment. Eight animals in both groups also completed the control experiment 1 week later. Histological stainings using vasopressin (VP) and vasoactive intestinal polypeptide (VIP) immunocytochemistry were used to check for remaining pieces of SCN tissue at the border of the lesion after finishing the physiological experiments, but in all 10 cases could confirm the completeness of the lesion. Typical examples of such a lesion and the histological stainings can be found in our previous papers [10], [16], [24]–[27]. In agreement with the findings in Experiment-1, BIC caused a significant hyperglycemia in the SHAM-lesioned animals, but in SCN-lesioned animals glucose responses during BIC administration did not differ from those on the control day (Fig. 3). Due to the large variability in the glucose responses of the BIC treated SCN-lesioned animals the effect of *Lesion* (i.e. BIC in SCN-lesioned versus BIC in SCN-sham animals) did not reach significance (p = 0.122), but the AUC for the glucose response to BIC was significantly smaller in the SCN-lesioned animals than that in the SHAM-lesioned animals (p = 0.041). In conclusion, ablation of the suprachiasmatic nuclei removes (or silences) the majority of the GABAergic inputs to the PVN that are responsible for the hyperglycemia induced by the PVN administration of the GABA-antagonist BIC.

### Experiment 3 : Daily changes in PVN neuronal activity and its effects on feeding-induced insulin responses

**Figure 4:**
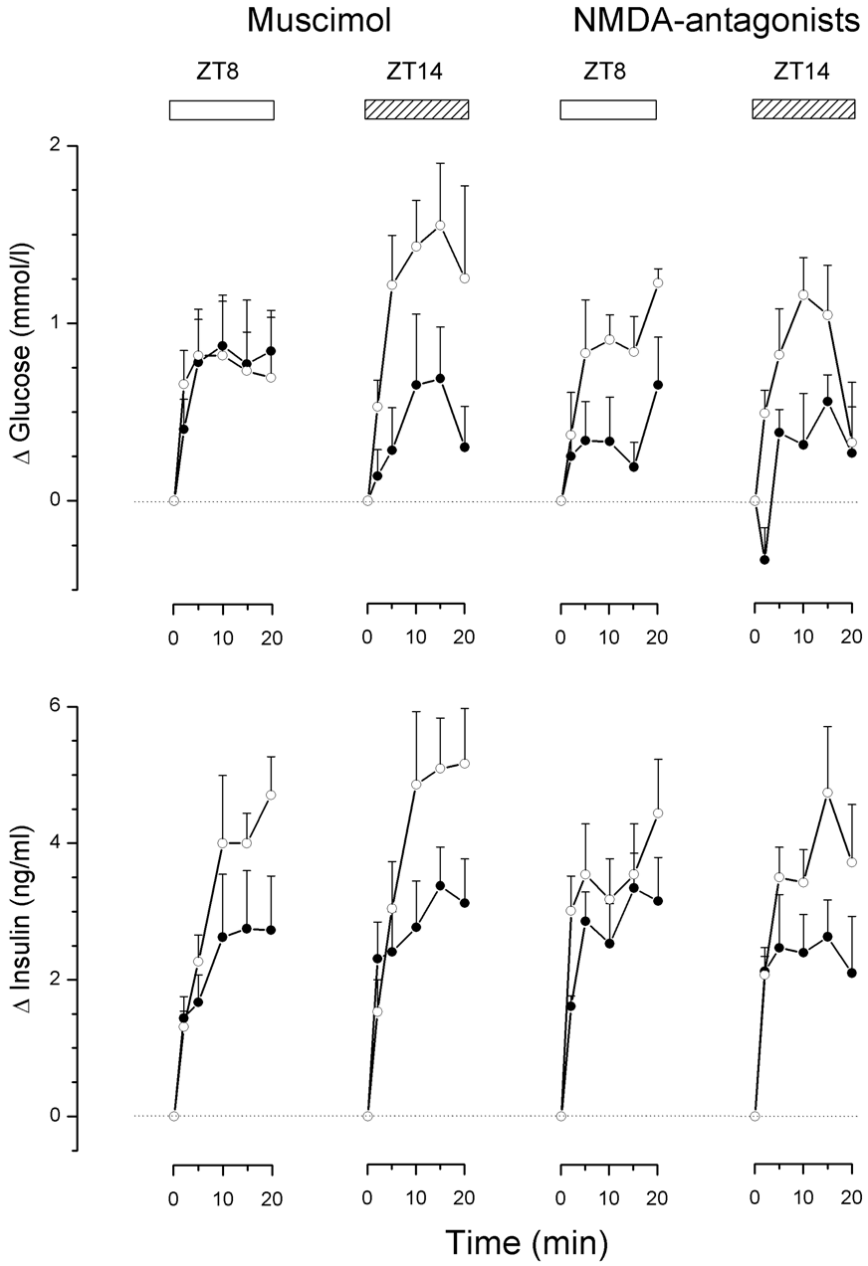
Changes in plasma glucose and plasma insulin concentrations during a scheduled meal at either ZT8 or ZT14 and the concurrent administration of Muscimol or a cocktail of NMDA-antagonists in the PVN (closed circles). Open circles show the results of the control experiment in the same animals one week later. Meal feeding caused significant increases of plasma glucose and plasma insulin concentrations on all occasions. Both Muscimol and treatment with the NMDA-antagonist cocktail caused a significant reduction of the glucose and insulin response during the ZT14, but not the ZT8, meal. These changes in glucose and insulin responses were observed despite matching of meal size during drug treatment and control meals. For details of statistical analysis see Tables S6 and S7.

Basal plasma glucose and hormone concentrations, i.e. just before opening the door in front of the food hopper and the start of the meal, are indicated in Table S3. PVN administration of MUS during feeding at ZT8 did not significantly affect the glucose and insulin responses, despite the somewhat smaller mean insulin increments during the experimental session (Fig. 4). On the other hand, enhancing GABAergic activity in the PVN during the ZT14 meal caused a significant decrease of the feeding-induced glucose response, as well as the insulin response (Table S4). Administration of the cocktail of NMDA antagonists in the PVN during the ZT8 meal did not significantly attenuate the glucose response, or the insulin response (Table S4). On the other hand, blockade of the NMDA receptors in the PVN during the ZT14 meal caused a significant decrement of both the glucose and insulin response (Table S4). In conclusion, PVN neuronal activity during the ZT14 meal (i.e. in the dark period), but not the ZT8 meal (i.e. during the light period), is necessary to elicit a complete glucose and insulin response.

## Discussion

Glutamate and GABA are the most abundant excitatory and inhibitory neurotransmitters in the central nervous system. The present study shows a pronounced time-of-day dependent hyperglycemic effect of the GABA-antagonist bicuculline, but not the glutamate receptor agonist NMDA, when administered in the paraventricular nucleus of the hypothalamus (PVN). Results in SCN-lesioned animals clearly show that this time dependency is derived from the biological clock situated in the hypothalamic suprachiasmatic nuclei (SCN). Combined with our previous liver-denervation experiments [23], the present results reveal a control mechanism for the daily rhythm in plasma glucose concentrations that very much resembles the one described earlier for the daily rhythm in plasma melatonin concentrations [11]. The SCN uses a continuously active glutamatergic projection and a rhythmically active GABAergic projection to control the activity of the sympathetic pre-autonomic neurons in the PVN. The scheduled feeding experiments provide the first evidence that also the parasympathetic pre-autonomic neurons in the PVN are under the control of a rhythmic GABAergic input. Contrary to the sympathetic pre-autonomic neurons, however, activation of the parasympathetic pre-autonomic neurons does not depend on afferent glutamatergic inputs from the SCN.

An alternative explanation for the strong time-of-day dependency of the hyperglycemic effect of BIC could be the well-known rhythm in hepatic glycogen stores with its acrophase at light onset [28]–[30]. Moreover, recent gene expression studies have revealed several hundreds of genes in the liver showing a circadian expression pattern, even during fasting conditions, including the well known core clock genes as well as those encoding several key enzymes involved in glucose metabolism [12]–[15]. Since the trough in hepatic glycogen stores coincides in time with the reduced hyperglycemic response to BIC (i.e. at ZT15) [31]–[33], we investigated whether a (almost) complete absence of hepatic glycogen stores (induced by 2 nights of food deprivation) would result in a decreased hyperglycemic effect of BIC. The results of the food-deprivation experiment were very clear. Although food-deprivation caused a much more profound reduction of hepatic glycogen stores than the daily light/dark-cycle (i.e. >90% versus ±30%, respectively), it had no effect whatsoever on the BIC-induced hyperglycemia (Fig. S1). The first evidence for an involvement of the suprachiasmatic nuclei in the hyperglycemic effect of BIC administered in the PVN was given in our previous study [23], when we showed that the administration of TTX in the SCN and DMH, but not in the PVN or VMH, caused an increase of plasma glucose concentrations. This mimicry of the hyperglycemic effect of BIC by silencing the SCN or DMH indicated that part of the GABAergic input to the PVN might be derived from the SCN and DMH. The present study adds two more pieces of evidence for the involvement of the SCN. Next to the time-of-day dependency of the BIC-induced hyperglycemia, the absence of a BIC-induced hyperglycemic effect in the SCN-lesioned animals provides a strong indication that a major part of the GABAergic input to the PVN (at least the part that is responsible for the BIC-induced hyperglycemia) is under control of the SCN. This could be either direct projections from GABAergic SCN neurons to the PVN or GABAergic projections to the PVN derived from SCN target areas. In the intact animal these afferent GABAergic inputs to the PVN from, for instance the subPVN and DMH, would be activated by the SCN during the light period. SCN lesions result in a “silencing” of these intermediate GABAergic neurons and, subsequently, in a lack of effect of BIC in the PVN. The present results thus clearly indicate that a major part of the GABAergic input to the PVN is under (in)direct control of the SCN-a situation which is clearly different from the GABAergic afferents to the PVN that are in control of the HPA-axis, as an SCN-lesion does not remove the stimulatory effect of BIC on the release of corticosterone [25] and Fig. S2.

Recently Bando et al [34] demonstrated that the parasympathetic part of the autonomic nervous system, too, is under a rhythmic control of the SCN. However, our previous study [23], as well as the first set of experiments in the present study, provided little evidence for a hypothalamic control of the parasympathetic pre-autonomic neurons in the PVN. None of the drugs produced a clear hypoglycemia as would be expected with an activation of the parasympathetic input to the liver [35], [36]. Profound changes in plasma insulin concentrations were not observed either, suggesting that clear effects on the parasympathetic system might only be detected when the system is stimulated. Indeed, whereas a daily rhythm in basal plasma insulin concentrations is small or non-existent [16], [37], [38], a pronounced daily modulation of the feeding-induced insulin responses has been described several times [39]–[41]. Therefore, we decided to investigate whether a modulation of the neuronal activity in the PVN during feeding would reveal clearer effects on the parasympathetic system (i.e. insulin release). The results of these experiments show that ±35% of the feeding-induced insulin response during the dark period is dependent on an activation of PVN neurons. Since the magnitude of the MUS and NMDA-antagonist effects was quite comparable, the main force for the activation of PVN neurons seems to be a glutamatergic input. On the other hand, the feeding-induced insulin response at ZT8 is not dependent upon neuronal activity in the PVN, since neither MUS nor the cocktail of NMDA-antagonists significantly changed the amount of insulin released. Silencing neuronal activity in the PVN during the ZT14 meal, with either MUS or NMDA-antagonists, also caused a ±65% reduction of the glucose response. Since the reduced glucose responses cannot be explained by smaller insulin responses, the inhibitory effects of MUS and the NMDA-antagonists on the plasma glucose responses indicate an increased nocturnal parasympathetic input-in this case an increased parasympathetic input to the gastro-intestinal tract that will shorten the transit time of food from the stomach to the intestines and will increase the absorption rate of glucose from the intestines. Indeed, both parameters also show a clear circadian rhythm mediated by the vagal innervation [42], [43].

**Figure 5:**
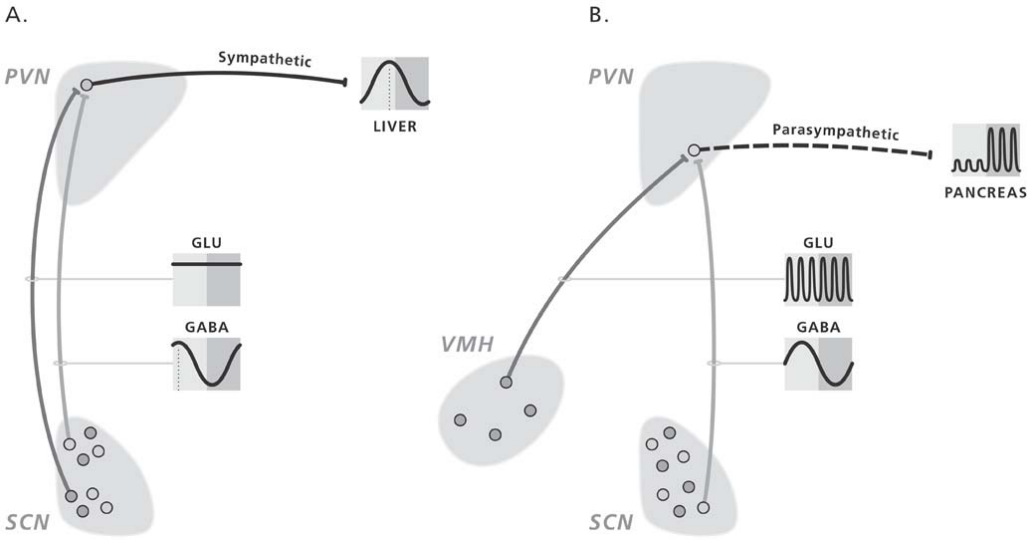
Schematic presentation of the daily activity pattern of hypothalamic populations of GABAergic and glutamatergic neurons implicated in the autonomic control of the daily rhythms in hepatic glucose production (left figure) and feeding-induced insulin release (right figure).

At present it is not clear where the glutamatergic input to the parasympathetic pre-autonomic PVN neurons originates, although the diurnal variation clearly indicates an involvement of the SCN. Strubbe et al [39] found increased glucose and insulin responses during daytime meals in SCN-lesioned animals, i.e. similar to the night time values in control animals. Similar data were provided by Yamamoto et al [40] after an oral glucose load. It was concluded that ablation of the SCN removes an inhibitory factor during the light period, and suggested that in the intact animal the SCN exerts an inhibitory effect on parasympathetic activity during the light period. Remarkably, Strubbe et al [39] also showed that prior treatment with atropine reduced the nighttime insulin and glucose responses to daytime values but hardly affected the daytime responses, i.e. very much similar to our MUS or NMDA-antagonist treatments. Could it be that like the sympathetic pre-autonomic neurons, the parasympathetic pre-autonomic neurons are also inhibited by a GABAergic input from the SCN during the daytime? Unfortunately we were unable to reveal such an inhibitory effect of GABA on the parasympathetic pre-autonomic neurons with BIC administration in the PVN, due to its strong activation of the sympathetic pre-autonomic neurons. The increased sympathetic input to the pancreas not only inhibits the release of insulin, but its strong hyperglycemic effect also obscures a possible hypoglycemic effect of an increased parasympathetic activity. On the other hand, the increased daytime glucose and insulin responses in the SCN-lesioned animals as described above indicate that the glutamatergic input to the parasympathetic pre-autonomic neurons is not coming from the SCN. Csaki et al. [44] showed that the PVN receives a vast amount of glutamatergic afferents, arising from a number of hypothalamic nuclei including the VMH and the arcuate nucleus [45]. Indeed electrical stimulation of the VMH increases glycogenolysis and glucose output from the liver mainly via the hepatic nerves [46]. More recently Tong et al [47] very nicely demonstrated that glutamate release from VMH neurons is necessary to prevent hypoglycemia (i.e. glutamate release from VMH neurons promotes glucose production). However, the direct projection from the VMH to the PVN appears to be rather sparse, whereas on the other hand massive projections have been described to the DMH and subPVN [48], [49]. Therefore, most of the VMH input to the pre-autonomic PVN neurons might be indirect through interneurons in the DMH and subPVN. Indeed, such an intermediate role for the DMH and subPVN was also proposed with regard to the SCN–PVN connection [50], [51]. We suggest that the (in)direct glutamatergic projections to the parasympathetic pre-autonomic neurons are activated, irrespective of the time-of-day, by viscerosensory stimuli propagated to the VMH via the nucleus of the solitary tract and parabrachial nucleus [52], [53] or by blood-borne signals acting through the arcuate nucleus [54]. The daily rhythm in parasympathetic PVN output then results from the rhythmic input of GABAergic SCN afferents (Fig. 5). Indeed, Decavel & VandenPol [55] showed a widespread convergence of GABAergic and glutamatergic afferents on PVN neurons.

In addition to probe placement, spread of the administered drugs to neighboring areas is always an important concern for the present type of studies. By comparing drug effects of the same drug applied in different hypothalamic nuclei we could calculate in a previous study that when applied by microdialysis the effective spread of BIC was ∼0.5 mm [23]. Thus, clearly with the current technique we are not able to differentiate between drug effects on the PVN proper and the subPVN. Therefore we cannot exclude that (some of) the effects involve indirect effects via intermediate neurons in the subPVN instead of direct projections to the pre-autonomic neurons. In fact, we recently found clear evidence for such a mechanism [56], albeit thus far only for the neuroendocrine PVN neurons. Our experiments were performed under L/D conditions thus it could be argued that the apparent temporal differences might be due to non-specific effects of light rather than a particular action of the SCN. Indeed, direct retinal projections to hypothalamic nuclei other than the SCN, including the (sub)PVN, have been demonstrated, be it to a much smaller extend [57], [58]. However, the results of the SCN lesion animals clearly show that a major part of the temporal difference in the hyperglycemic response to BIC is derived from SCN projections. As the temporal differences in the feeding-induced insulin response were also abolished by SCN removal [39], it seems very unlikely that the current results can be explained by non-specific effects of light. The SCN lesion experiments also exclude that the presently found temporal differences are due to diurnal differences in feeding behavior and/or locomotor activity. Moreover, the animals were either not eating during the experiment or fed a similar sized meal.

Next to the autonomic nervous system also circulating epinephrine, glucagon and corticosterone are major players in the regulation of plasma glucose [59], [60]. Moreover, GABA antagonism in the PVN also results in robust increases in plasma epinephrine [61], [62], as well as glucagon and corticosterone [23]. However, our denervation studies clearly show that these hormonal changes cannot be the sole explanation for the changes in hepatic glucose production observed after the hypothalamic infusions [23], [63]. Also in the current study there was no correlation between the drug-induced changes in plasma glucose (Fig. 2) and plasma corticosterone (Fig. S3).

With regard to the circadian control of the autonomic nervous system the picture that emerges from the present data and our previous experiments is one that shows an important role for a GABAergic-glutamatergic switch, not only with respect to the sympathetic branch but also where it concerns the parasympathetic branch. The different timing of the daily peak in melatonin release (i.e. ZT16–22) and hepatic glucose production (i.e. ZT10–14) indicates that the GABAergic efferents from the SCN differentiate between the pre-autonomic neurons that control the sympathetic input to the pineal and the neurons that control the sympathetic input to the liver. In view of this highly differentiated somatotopic organization it is not surprising that SCN efferents are also able to differentiate between sympathetic and parasympathetic pre-autonomic neurons. Indeed, using a combination of double retrograde viral tracing and selective organ denervation, we found neuro-anatomical evidence for a somatotopic organization in the SCN [64], as well as for the maintenance of a segregation of sympathetic and parasympathetic neurons up to the level of the SCN [65].

In conclusion, the daily rhythm in the activity of the pre-autonomic neurons is predominantly determined by somatotopically organized GABAergic SCN outputs that inhibit selective groups of pre-autonomic neurons at specific times of the L/D-cycle. The abundant presence of GABAergic neurons in the SCN nicely fits with such a prominent role for GABAergic SCN efferents [66], [67]. On the other hand, only a minor part of the rhythmic activity in pre-autonomic neurons seems to depend on glutamatergic efferents from the SCN. Finally, the prominent circadian rhythmicity of a number of peptidergic SCN transmitters [68], [69] and the consistent co-localization of small fast-acting transmitters such a GABA and glutamate with hypothalamic peptides [70], also within the SCN [67], [71], suggests an important modulatory role for the SCN peptides on its final output, despite the pronounced GABAergic and glutamatergic effects described above.

## Materials and Methods

### Animals

Male Wistar rats (Harlan, The Netherlands) were housed at a room temperature of 21±1 C with a 12-h light/dark (L/D) cycle (lights on at 07.00 h). For experiments performed during the dark period, animals were housed in a reversed L/D-cycle with lights on at 19.00 h. Animals were allowed to adapt to the new environment for 2 weeks (or 6 weeks in case of housing in a reversed L/D-cycle) before the first experiments. Animals were kept with 4–6 animals per cage until one week before surgery, at which time they were transferred to individual cages (25×25×35 cm). Food and water were available *ad libitum*, except during experimental sessions, when only water was available. Animals weighed between 300–350 g at the time of experiments. Rats were anaesthetized using a mixture of Hypnorm (0.05 ml/100 g body weight, i.m.) and Dormicum (0.04 ml/100 g body weight, s.c.). All of the following experiments were conducted under the approval of the Animal Care Committee of the Royal Netherlands Academy of Arts and Sciences.

### Surgical procedures

#### Jugular venectomy and intracerebral microdialysis probes

Experimental animals destined to undergo infusion and blood sampling studies were fitted with bilateral microdialysis probes and an intra-atrial silicone catheter through the right jugular vein when the body weight had reached 300 g, as described previously [23]. After surgery, rats were allowed to recover for at least 10 days to allow complete reinstatement of circadian rhythms [72]. During experiments, the animals were permanently connected to the blood-sampling and microdialysis lines, as described previously [23]. The microdialysis probes were stereotaxically implanted directly lateral to the PVN (stereotaxic coordinates with flat skull: 1.8 mm posterior to bregma, 2.0 lateral from the midline, 7.9 mm below the brain surface, and 10° angled to the sagittal plane).

#### SCN-lesions

A total of 36 rats (180–200 g) were operated upon in order to ensure a sufficient number of effectively lesioned animals. For the SCN lesion procedure animals were mounted with their heads in a David Kopf stereotact (Tujunga, CA) with the toothbar set at +5.0 mm, and sustained a bilateral lesion of their SCN (coordinates 1.4 mm rostral to bregma, 1.1 mm lateral to the midline and 8.3 mm below the brain surface) using bilateral lesion electrodes, 0.2 mm in diameter, that were heated to 85°C for 1 minute (lesion generator, Radionics, Burlington, MA). This temperature/duration combination was found empirically to result in lesion large enough to eliminate the SCN, but small enough to leave surrounding hypothalamic structures, such as the PVN and supraoptic nucleus, intact. Drawback of minimizing the lesion size, however, is the limited yield of completely lesioned animals (∼30%). In order to restrict the number of (false positive) animals to be operated upon an initial screening is made by measuring their diurnal water intake. After a 2-week recovery period the effectiveness of the SCN-lesions was checked by measuring the animals' drinking behavior during a 3-week period [10], [27]. By this method 11 animals were selected as being completely arrhythmic. SHAM-lesioned animals were treated in exactly the same way as the SCN-lesioned animals, except for the fact that the lesion electrodes were not heated. In the 6^th^ week after the operation groups of effectively SCN-lesioned animals and SHAM-lesioned animals were operated upon as described above.

#### Physiological experiments

Ringer's perfusion (3 µl/min) was started 2 hours before the start of the 2-h drug infusion period and lasted for 2 more h after the change back to Ringer = s. The 2-h drug infusion periods were initiated at ZT5 or ZT15. Blood samples (0.6 ml) were taken 30 min before (t = −30) and 0, 30, 60, 120 and 180 min after the start of the drug infusion. The following neurotransmitter (ant)agonists were administered to the PVN: muscimol (MUS; a potent GABA_A_ receptor agonist; 100 µM), bicucilline (BIC; a GABA_A_ receptor antagonist; 100 µM), NMDA (a glutamate receptor agonist; 100 µM), and MK801 (a potent, selective and non-competitive NMDA receptor antagonist; 100 µM). In the scheduled feeding experiments (see below) a cocktail of NMDA antagonists was used. This cocktail consisted of MK801 (100 µM), AP5 (500 µM), and DNQX (100 µM). All drug concentrations were chosen on basis of our own previous microdialysis experiments (BIC, MUS, NMDA and MK801 [10], [23] or other studies employing reverse microdialysis to administer these drugs (AP5 [73] and DNQX [74]). In principle 1 week later all animals participated in a 2^nd^ session without perfusion of the microdialysis probes, but with blood samples taken at the appropriate time points.

For the scheduled feeding experiment, rats were entrained to a scheduled feeding regimen very much similar to our previously published method [16], [41], [75]. However, to ensure a more robust glucose and insulin response (in view of the somewhat more arousal evoking environment of hypothalamic manipulations), rats were entrained to four instead of six meals spread equally over the L/D cycle. In a pilot experiment we assured that this 4-meals-a-day schedule also evoked a daily rhythm in feeding-induced insulin responses. Meals started at ZT2, ZT8, ZT14, and ZT20. The access to food was 15 minutes for the day-time and 12 minutes for the night-time meals. The rats were given three weeks to adapt to the feeding schedule.This regular feeding schedule was maintained until the end of the experiments.

#### Histology

When the experimental protocol was completed animals were anaesthetized with CO_2_/O_2_ and decapitated. Brains were subsequently removed, blocked, frozen, sectioned (40 µm) through the hypothalamus, and stained with cresyl violet. In the case of SCN-lesions the sections were processed for vasopressin and VIP immunocytochemistry.

#### Analytical methods

Blood samples were immediately chilled on ice in tubes containing a 10 µl solution of 2.5% EDTA+10% benzamidine hydrochloride (BDH) and centrifuged at 4 C. Plasma was then stored at −80 C until further analysis. Plasma glucose concentrations were determined using a Glucose/GOD-Perid method (Boehringer Mannheim, Mannheim, Germany). Plasma immunoreactive insulin and corticosterone concentrations were measured using radioimmunoassay kits (LINCO Research Inc., Missouri, USA and ICN Biomedicals, Costa Mesa, CA, respectively). All samples were assayed in duplicate. Hepatic glycogen content was measured as described previously [76].

#### Statistical analysis

We evaluated the kinetics of the plasma hormone and glucose concentrations as a consequence of the hypothalamic infusions (and the food intake) by using the increments of their plasma concentrations compared with the t = 0 value. The significance of the infusion- (or feeding-)induced variations in plasma glucose and hormone values was assessed using a multivariate analysis of variance (MANOVA) with repeated measures with *Sampling* (6 levels) and *Treatment* (2 levels, i.e. experimental day versus control day) as the repeated within-animal factors and, depending on the experiment, *ZT-time* (2 levels) or *Lesion* (2 levels) as a between-animal factor. MANOVA was followed by post-hoc testing (Fischer's least significant difference (L.S.D.)) if significant effects of *Treatment*, *ZT-time*, *Food*, *Lesion*, *or Interaction* were detected. In Experiment-2 (SCN lesions) we also used the area under the curve (AUC) as a reflection of the overall change in the 180 min (post)infusion period to evaluate the plasma glucose changes. Also the AUC was calculated from the incremental data. Within- and between-group differences in (basal) glucose and hormone concentrations were analyzed using the Student's *t*-test for paired and unpaired samples, respectively. Statistical significance was set at p<0.05 for a two-tailed test.

## Supporting Information

Figure S1 Supplemental Fig. 1 Changes in plasma glucose, glucagon and corticosterone concentrations during a 2-h Bicuculline administration in the PVN of ad libitum fed (closed circles), 16-h food-deprived (closed triangles in the left column) or 40-h food deprived animals (closed triangles in the right column). Bicuculline was administered from ZT5–ZT7. Open symbols show the results of the control experiment in the same animals one week later (i.e. with food-deprivation, but without Bicuculline administration). In food-deprived animals food was removed at ZT12 on the day before (∼16-h food deprivation) or 2 days before the Bicuculline experiment (∼40-h food deprivation). In ad libitum fed animals food was only absent during the experiment, i.e. ZT3–ZT8. For details of statistical analysis see Tables S4 and S5.
(8.73 MB TIF)

Figure S2 Supplemental Fig. 2 Changes in plasma corticosterone concentrations during a 2-h administration of Bicuculline in the PVN of SCN-lesioned (closed triangles) and sham-lesioned animals (closed circles). Open symbols show the results of the control experiment in the same animals one week later. Notwithstanding the profound reduction of the Bicuculline induced hyperglycemia in the SCN-lesioned animals (Fig. 3) the corticosterone responses in these animals are still intact. During the control day the SCN-lesioned animals showed even a somewhat higher (stress) response (Lesion, p = 0.024 and Sample×Lesion, p = 0.007).
(7.24 MB TIF)

Figure S3 Supplemental Fig. 3 Changes in plasma corticosterone concentrations during a 2-h administration of NMDA, the NMDA antagonist MK801, Bicuculline, or Muscimol in the PVN at either ZT5 or ZT15. The results of the ZT5 experiments are represented by circles and those of the ZT15 experiments by triangles. Filled symbols indicate the effect of the drug, whereas open symbols show the results of the control experiment in the same animals one week later.
(5.63 MB TIF)

Table S1 (0.04 MB DOC)

Table S2 (0.08 MB DOC)

Table S3 (0.08 MB DOC)
