# Supplementary material for: Circadian Control of the Daily Plasma Glucose Rhythm: An Interplay of GABA and Glutamate
Source: PLoS One. 2008 Sep 15;3(9):e3194. doi: 10.1371/journal.pone.0003194 (PMC2527681; doi:10.1371/journal.pone.0003194)
Supplement: Table S1 — (0.04 MB DOC) [file pone.0003194.s004.doc]

**Table S1** Statistical analysis of the glucose responses during the 2-h PVN administration of GABA and glutamate (ant)agonists in either the light- or dark period

­­­­­­­­­­­­­­­­­­­­­_________________________________________________

**ZT5 ZT15**

**NMDA**

*Sample* 0.276 <0.001

*Treatment* 0.008 0.009

*Interaction* 0.055 0.066

n=7 n=9

**MK801**

*Sample* 0.408 <0.001

*Treatment* 0.087 0.580

*Interaction* 0.001 0.965

n=7 n=6

**Bicuculline**

*Sample* 0.055 <0.001

*Treatment* 0.002 0.867

*Interaction* 0.016 0.538

n=7 n=7

**Muscimol**

*Sample* 0.607 0.046

*Treatment* 0.340 0.131

*Interaction* 0.444 0.101

n=10 n=6

ZT5 and ZT15 *p*-values indicate the ANOVA results for the comparison between the experimental day (i.e. with drug administration via the microdialysis probes) and the control day ~1 week later in the same group of animals (i.e. with blood sampling, but without microdialysis). The ZT5 vs. ZT15 *p*-values indicate the ANOVA results for the comparison between drug treatments at ZT5 and ZT15 (i.e. different groups of animals).
