# Supplementary material for: Circadian Control of the Daily Plasma Glucose Rhythm: An Interplay of GABA and Glutamate
Source: PLoS One. 2008 Sep 15;3(9):e3194. doi: 10.1371/journal.pone.0003194 (PMC2527681; doi:10.1371/journal.pone.0003194)
Supplement: Table S2 — (0.08 MB DOC) [file pone.0003194.s005.doc]

**Table S2** Basal glucose and hormone concentrations at the start of the ZT5 and ZT15 experiments

|  |  | **ZT5** |  |  |  | **ZT15** |  |  |  |
| --- | --- | --- | --- | --- | --- | --- | --- | --- | --- |
|  | n | Drug | Control | *p* | n | Drug | Control | *p* | *p** |
| **BIC** |  |  |  |  |  |  |  |  |  |
| Glucose | 7 | 6.4 ± 0.5 | 6.9 ± 0.3 | 0.122 | 7 | 6.3 ± 0.1 | 6.3 ± 0.2 | 0.836 | 0.962 |
| Corticosterone | 7 | 44 ± 21 | 22 ± 6 | 0.338 | 5 | 96 ± 22 | 59 ± 17 | 0.189 | 0.066 |
| Insulin | 7 | 2.4 ± 0.3 | 1.9 ± 0.2 | 0.157 | 3 | 0.9 ± 0.1 | 0.7 ± 0.1 | 0.421 | 0.019 |
|  |  |  |  |  |  |  |  |  |  |
| **NMDA** |  |  |  |  |  |  |  |  |  |
| Glucose | 7 | 5.9 ± 0.5 | 6.8 ± 0.4 | 0.044 | 9 | 6.7 ± 0.2 | 6.7 ± 0.2 | 0.989 | 0.174 |
| Corticosterone | 7 | 30 ± 13 | 17 ± 5 | 0.341 | 4 | 41 ± 12 | 98 ± 32 | 0.068 | 0.557 |
| Insulin | 7 | 2.0 ± 0.4 | 2.6 ± 0.7 | 0.419 | 6 | 1.0 ± 0.1 | 1.6 ± 0.2 | 0.024 | 0.052 |
|  |  |  |  |  |  |  |  |  |  |
| **MUS** |  |  |  |  |  |  |  |  |  |
| Glucose | 10 | 5.8 ± 0.4 | 6.7 ± 0.3 | 0.009 | 6 | 6.7 ± 0.1 | 6.6 ± 0.2 | 0.771 | 0.091 |
| Corticosterone | 10 | 19 ± 12 | 20 ± 5 | 0.911 | 6 | 205 ± 54 | 119 ± 49 | 0.135 | 0.000 |
| Insulin | 10 | 2.1 ± 0.2 | 3.0 ± 0.6 | 0.104 | 4 | 1.6 ± 0.2 | 1.1 ± 0.2 | 0.129 | 0.254 |
|  |  |  |  |  |  |  |  |  |  |
| **MK801** |  |  |  |  |  |  |  |  |  |
| Glucose | 7 | 6.4 ± 0.3 | 6.7 ± 0.3 | 0.278 | 6 | 6.2 ± 0.2 | 7.0 ± 0.3 | 0.018 | 0.599 |
| Corticosterone | 7 | 74 ± 22 | 35 ± 8 | 0.091 | 3 | 14 ± 36 | 174 ± 50 | 0.289 | 0.161 |
| Insulin | 4 | 1.5 ± 0.2 | 2.9 ± 0.4 | 0.026 | 5 | 1.3 ± 0.3 | 2.1 ± 0.4 | 0.310 | 0.593 |
|  |  |  |  |  |  |  |  |  |  |
| **All** |  |  |  |  |  |  |  |  |  |
| Glucose | 31 | 6.1 ± 0.2 | 6.8 ± 0.2 | 0.000 | 28 | 6.5 ± 0.1 | 6.7 ± 0.1 | 0.241 | 0.088 |
| Corticosterone | 31 | 39 ± 9 | 23 ± 3 | 0.051 | 18 | 123 ± 24 | 107 ± 21 | 0.498 | 0.000 |
| Insulin | 28 | 2.1 ± 0.2 | 2.6 ± 0.3 | 0.065 | 18 | 1.2 ± 0.1 | 1.5 ± 0.2 | 0.242 | 0.000 |

The *p*-value indicates the results of the paired Student’s *t*-test for the comparison between the day of drug treatment and the control day. The *p**-value indicates the results of the unpaired Student’s *t*-test for the comparison between the drug treatment days of the ZT5 and ZT15 experiments.
