# Supplementary material for: Circadian Control of the Daily Plasma Glucose Rhythm: An Interplay of GABA and Glutamate
Source: PLoS One. 2008 Sep 15;3(9):e3194. doi: 10.1371/journal.pone.0003194 (PMC2527681; doi:10.1371/journal.pone.0003194)
Supplement: Table S3 — (0.08 MB DOC) [file pone.0003194.s006.doc]

**Table S3** Meal sizes and basal glucose and insulin concentrations during the ZT8 and ZT14 scheduled feeding experiments

|  | n | **Drug** | **Control** | *p* | *p** |
| --- | --- | --- | --- | --- | --- |
| **Glucose** |  |  |  |  |  |
| *Muscimol* |  |  |  |  |  |
| ZT8 | 8 | 5.8 ± 0.2 | 5.8 ± 0.1 | 0.831 |  |
| ZT14 | 10 | 6.3 ± 0.1 | 5.9 ± 0.2 | 0.200 | 0.036 |
|  |  |  |  |  |  |
| *NMDA-anta* |  |  |  |  |  |
| ZT8 | 11 | 6.3 ± 0.2 | 6.1 ± 0.2 | 0.530 |  |
| ZT14 | 9 | 5.9 ± 0.1 | 5.6 ± 0.2 | 0.112 | 0.295 |
|  |  |  |  |  |  |
|  |  |  |  |  |  |
| **Insulin** |  |  |  |  |  |
| *Muscimol* |  |  |  |  |  |
| ZT8 | 8 | 1.0 ± 0.2 | 0.9 ± 0.1 | 0.568 |  |
| ZT14 | 10 | 1.8 ± 0.3 | 1.5 ± 0.4 | 0.305 | 0.064 |
|  |  |  |  |  |  |
| *NMDA-anta* |  |  |  |  |  |
| ZT8 | 11 | 1.1 ± 0.1 | 1.4 ± 0.2 | 0.310 |  |
| ZT14 | 9 | 2.5 ± 0.4 | 1.8 ± 0.4 | 0.121 | 0.001 |
|  |  |  |  |  |  |
|  |  |  |  |  |  |
| **Meal size** |  |  |  |  |  |
| *Muscimol* |  |  |  |  |  |
| ZT8 | 8 | 4.6 ± 0.4 | 4.1 ± 0.6 | 0.503 |  |
| ZT14 | 10 | 4.3 ± 0.3 | 4.0 ± 0.3 | 0.230 | 0.569 |
|  |  |  |  |  |  |
| *NMDA-anta* |  |  |  |  |  |
| ZT8 | 11 | 4.1 ± 0.4 | 4.6 ± 0.3 | 0.119 |  |
| ZT14 | 9 | 3.7 ± 0.2 | 3.9 ± 0.2 | 0.376 | 0.394 |

The *p*-value indicates the results of the paired Student’s *t*-test for the

comparison between the day of drug treatment and the control day. The

*p**-value indicates the result of the unpaired Student’s *t*-test for the comparison

of the drug treatment day between the ZT8 and ZT14 experiment.
